# Correlations between gut microbiota and serum metabolomics in patients with neurogenic rosacea

**DOI:** 10.1186/s12866-025-04173-3

**Published:** 2025-07-17

**Authors:** Min Li, Jian Fu, Jinyu Wei, Mei Wan, Ling Li, Minmin Kong, Shuguang Chen, Lian Zhang, Aiai Xia, Li Tang, Fei Hao, Jian Li

**Affiliations:** 1 Department of Dermatology, Chinese People’s Liberation Army Western Theater Command General Hospital, Chengdu, China; 2 Department of Urology, Southwest Hospital, First Affiliated Hospital of Army Medical University, Chongqing, China; 3 Department of Dermatology, Southwest Hospital, First Affiliated Hospital of Army Medical University, Gaotanyan Main Street 30, Shapingba District, Chongqing, 400038 China; 4 The Third Affiliated Hospital of Chongqing Medical University, Chongqing, China

**Keywords:** Neurogenic rosacea, Metabolomics, Gut microbiota, Gut-skin axis

## Abstract

This study aimed to investigate the association between serum metabolomic profiles and the gut microbiota in patients with neurogenic rosacea. We analyzed the serum metabolites and gut microbial composition in 20 patients with neurogenic rosacea and 14 healthy controls. Metabolomic analysis identified 56 differentially abundant metabolites (DAMs), with benzene and substituted derivatives, carboxylic acids and derivatives, and organophosphates being among the most enriched categories. Gut microbiota analysis revealed significant dysbiosis in patients with neurogenic rosacea, characterized by an increased abundance of *Lachnospiraceae*, *Veillonellaceae*, and *Clostridiaceae*, and decreased levels of *Prevotellaceae* and *Bifidobacteriaceae.* Spearman’s correlation analysis demonstrated significant relationships between specific serum metabolites and gut microbial taxa, including strong positive correlations between 3,4-dihydroxyphenylacetic acid and both *Bifidobacterium* (r = 0.7307, padj = 9.25e-07) and *Lactobacillus* (r = 0.6898, padj = 6.39e-06). These findings highlight the complex interactions between metabolic dysregulation and gut microbial imbalances in neurogenic rosacea, suggesting a potential role for the gut-skin axis in its pathophysiology. This study paves the way for future therapeutic strategies targeting both metabolic and microbial dysbiosis to manage neurogenic rosacea.

**Supplementary Information:**

The online version contains supplementary material available at 10.1186/s12866-025-04173-3.

## Introduction

Neurogenic rosacea is a chronic inflammatory skin disorder characterized by persistent facial erythema, flushing, and symptoms related to sensory disturbances such as burning and stinging sensations [1]. It primarily affects adults, particularly women, and is often exacerbated by various environmental and lifestyle factors including emotional stress, temperature fluctuations, sun exposure, and dietary triggers [2]. Despite its prevalence, the underlying mechanisms of neurogenic rosacea remain poorly understood, posing challenges for its effective diagnosis and management.

Emerging evidence suggests that rosacea may be closely linked to dysregulation in both the immune response and the peripheral nervous system [3, 4]. Neurogenic inflammation plays a central role in the pathophysiology of rosacea, evidenced by increased sensory nerve interactions with blood vessels and mast cells, as well as early activation of mediators and receptors involved in neurovascular and neuroimmune communication [5]. Furthermore, recent studies have begun to investigate the potential role of the gut-skin axis in dermatological diseases, highlighting how systemic inflammation and gut microbiota alterations may influence skin health [6–8].

Gut microbiota, a complex and dynamic community of microorganisms residing in the gastrointestinal tract, has been implicated in various inflammatory and metabolic disorders [9, 10]. Dysbiosis, defined as an imbalance in the composition and function of the gut microbiota, has been linked to several skin conditions, including acne, atopic dermatitis, psoriasis, and chronic urticaria [11, 12]. The interplay between gut microbiota and skin disorders may occur through several mechanisms, including modulation of immune responses, production of metabolites, and direct influences on skin barrier function [13–15].

In addition to microbial dysbiosis, alterations in the serum metabolomic profiles have garnered attention in recent years. Metabolomics provides a comprehensive approach to understanding biochemical changes associated with diseases by examining small-molecule metabolites in biological samples [16]. In the context of neurogenic rosacea, the identification of specific metabolites could yield valuable insights into the disease's underlying metabolic pathways and contribute to the development of targeted therapeutic strategies.

This study aimed to investigate the metabolic and microbial profiles of patients with neurogenic rosacea compared to those of healthy controls, focusing on identifying potential biomarkers and understanding the interplay between serum metabolites and gut microbiota. By elucidating these connections, we hope to enhance our understanding of the pathophysiology of neurogenic rosacea and to identify novel therapeutic targets that may improve patient outcomes.

## Methods

### Study subjects

From July 2022 to October 2023, participants were recruited at the Southwest Hospital, First Affiliated Hospital of Army Medical University, for a study investigating neurogenic rosacea. This study was approved by the Ethics Committee of the First Affiliated Hospital of the Army Medical University (approval number (A)KY2023192. Eligible patients were aged 18–50 years and diagnosed with neurogenic rosacea, characterized by persistent facial erythema and neurologically related symptoms such as burning and stinging sensations. Symptoms were required to be exacerbated by factors such as emotional stress, temperature fluctuations, sun exposure, and consumption of spicy foods. All participants provided written informed consent prior to enrollment.

Exclusion criteria for the neurogenic rosacea patients included known allergies to study treatments; serious cardiovascular, pulmonary, hepatic, or renal conditions; recent use of medications that may affect rosacea symptoms (within the past month); as well as pregnancy, lactation, or significant mental or neurological disorders. The healthy control group consisted of age- and gender-matched individuals without any history of dermatological or systemic diseases and who did not meet any of the exclusion criteria applied to the patient group.

### Plasma sample collection

Peripheral blood samples were collected from both patients and healthy subjects using ethylenediaminetetraacetic acid (EDTA) anticoagulant vacuum tubes early in the morning. The samples were gently mixed by inverting the tubes several times to prevent clotting. Subsequently, the blood was centrifuged at 3500 × g for 10 min at 4 °C. The resulting plasma was transferred into new centrifuge tubes and rapidly frozen in liquid nitrogen. All separated plasma samples were subsequently stored at −80 °C for future analysis.

### Sample preparation

Serum samples were thawed at 4 °C and vortexed for 1 min to ensure homogeneity. A 200 µL aliquot was transferred into a 2 mL centrifuge tube. Then, 400 µL of pre-chilled methanol (stored at − 20 °C) was added, and the mixture was vortexed for 1 min. The samples were centrifuged at 12,000 rpm for 10 min at 4 °C, and the resulting supernatant was transferred to a new 2 mL tube. The samples were then dried using a vacuum concentrator (SpeedVac). The dried residue was reconstituted in 150 µL of 4 ppm 2-chloro-l-phenylalanine in 80% methanol, filtered through a 0.22 μm membrane, and transferred to autosampler vials for LC–MS/MS analysis [17].

### Liquid Chromatography-tandem Mass Spectrometry (LC–MS/MS) analysis

LC–MS/MS analysis was performed using a Vanquish UHPLC system (Thermo Fisher Scientific) coupled with a Q Exactive HFX mass spectrometer (Orbitrap MS, Thermo Fisher Scientific). Chromatographic separation was achieved using an UPLC BEH Amide column. All chromatographic and mass spectrometric parameters were processed following standard procedures by the manufacturer, including optimal column temperature, flow rate, gradient elution, and ionization conditions, ensuring reproducibility and accuracy of metabolite detection [18, 19].

### Data processing and multivariate analysis

The raw data from LC–MS/MS analysis were first converted to the mzXML format using MSConvert in the ProteoWizard software package (version 3.0.8789) [20]. The converted data were then processed using XCMS [21] for feature detection, retention time correction, and alignment. Metabolites were identified based on accurate mass (with a tolerance of < 30 ppm) and MS/MS data, which were matched against several databases including HMDB [22], MassBank [23], LipidMaps [24], mzCloud [25], and KEGG [26]. To correct for systematic bias, robust LOESS signal correction (QC-RLSC) [27] was applied for data normalization. Following normalization, only ion peaks with a relative standard deviation (RSD) of less than 30% in quality control (QC) samples were retained to ensure accurate metabolite identification.

Multivariate data analysis and modeling were conducted using Ropls [28]. After scaling the data, models were built using Principal Component Analysis (PCA), Partial Least Squares Discriminant Analysis (PLS-DA), and Orthogonal Partial Least Squares Discriminant Analysis (OPLS-DA). Metabolic profiles were visualized using score plots, where each point represented a sample. Corresponding loading plots and S-plots were generated to provide insights into the metabolites responsible for sample clustering. To assess potential overfitting, all the models were validated using permutation tests. The descriptive performance of the models was evaluated using the cumulative R2X and R2Y values (with a perfect model with R2X(cum) = 1 and R2Y(cum) = 1), while the predictive performance was assessed using the cumulative Q2 value (with a perfect model having Q2(cum) = 1). In the permutation test, the permuted model should not be able to predict class separation; thus, the R2 and Q2 values at the Y-axis intercept must be lower than those of the nonpermuted model.

OPLS-DA was used to identify discriminating metabolites based on the Variable Importance on Projection (VIP) scores. To determine the significant contributors to classification, statistical criteria such as P value, VIP score, and fold change (FC) were applied. Metabolites with a P value < 0.05 and VIP score > 1 were considered statistically significant.

### Fecal sample collection and analysis

Fecal samples were collected from adult human participants, immediately flash-frozen in liquid nitrogen, and stored at − 80 °C until DNA extraction. Microbial genomic DNA was extracted from each fecal sample (250–500 mg) using the E.Z.N.A.® Soil DNA Kit (Omega Bio-Tek, Norcross, GA, USA), following the manufacturer’s instructions. The concentration and purity of the extracted DNA were assessed using a NanoDrop 2000 UV–Vis spectrophotometer (Thermo Scientific, Wilmington, MA, USA) and 1% agarose gel electrophoresis.

### 16S rRNA sequencing

Sequencing was performed on the Illumina Hiseq2500 platform using a paired-end strategy with a read length of 2 × 250 bp. An average of 53,218 high-quality reads per sample was obtained (range: 49,882–56,703). The V3–V4 region of the bacterial 16S rRNA gene was amplified using primers 341 F (5′-CCTAYGGGRBGCASCAG-3′) and 806R (5′-GGACTACNNGGGTATCTAAT-3′). PCR amplification used Pfu high-fidelity DNA polymerase and was conducted for 27 cycles under the following conditions: 95 °C for 3 min; 27 cycles of 95 °C for 30 s, 55 °C for 30 s, and 72 °C for 45 s. Sequencing was conducted using standard Illumina protocols with dual-index barcoding. Library preparation included end repair, A-tailing, adapter ligation, and limited-cycle PCR enrichment. Final libraries were quantified using PicoGreen and analyzed for fragment size with a Bioanalyzer.

### Sequencing data analysis

Paired-end reads were merged using FLASH (v1.2.7) with a minimum overlap of 10 bp and a maximum mismatch ratio of 10%. Low-quality sequences and chimeras were filtered out using QIIME (version 2022.2) with a minimum Phred quality score threshold of 20. High-quality reads were clustered into operational taxonomic units (OTUs) at a 97% similarity threshold using USEARCH (v7.1). Taxonomic classification was performed using the SILVA 138 database via a naïve Bayesian classifier. OTU abundance plots were generated to reflect gut microbial diversity based on the relative abundance of taxa across samples.

To assess within-sample diversity, α-diversity indices including Chao1, Shannon, and Simpson were calculated. For between-group comparisons, β-diversity was assessed using principal coordinates analysis (PCoA) and non-metric multidimensional scaling (NMDS). Linear Discriminant Analysis (LDA) was performed using standardized microbial abundance data, and LDA projections were used to visualize microbial community separation between neurogenic rosacea patients and healthy controls.

Furthermore, the Linear Discriminant Analysis Effect Size (LEfSe) algorithm was used to identify taxa with significantly different abundance between groups. Default parameters were applied, and taxa with an LDA score > 4 were considered differentially enriched biomarkers [29].

All scripts, analysis pipelines, and parameter settings used for bioinformatics analysis are publicly available at: https://github.com/limin0214/neurogenic-rosacea-pipeline.

### Statistical analysis

The measurement data are expressed as the mean ± standard deviation, while enumeration data are represented as percentages. The χ^2^ test was used for inter-group comparisons. Statistical significance was set to indicate statistically significant differences.

## Results

### Patient characteristics

A total of 34 participants were included in this study, comprising 20 patients with neurogenic rosacea and 14 healthy controls. There were no significant differences in sex, age, weight, height, or BMI between neurogenic rosacea patients and healthy controls. Participants’ characteristics are presented in Table 1.

**Table 1:** Characteristics of patients with neurogenic rosacea and healthy subjects

Characteristics	Patients with neurogenic rosacea	Healthy controls	*P* value
Number	20	14	NA
Sex,male/female	2/18	1/13	0.635
Age (y), mean±SD	29.30±9.97	33.93±6.75	0.071
Height (cm), mean±SD	160.55±5.35	159.29±4.01	0.460
Weight (kg), mean±SD	53.6±7.21	53.14±3.18	0.826
BMI (kg/m²),mean±SD.	20.73±2.32	20.96±1.43	0.744

*NA *Not available, *SD *Standard deviation, *NS *Not significant, *BMI *Body mass index

### Serum metabolomic profiles and differential metabolites

**Fig. 1:**
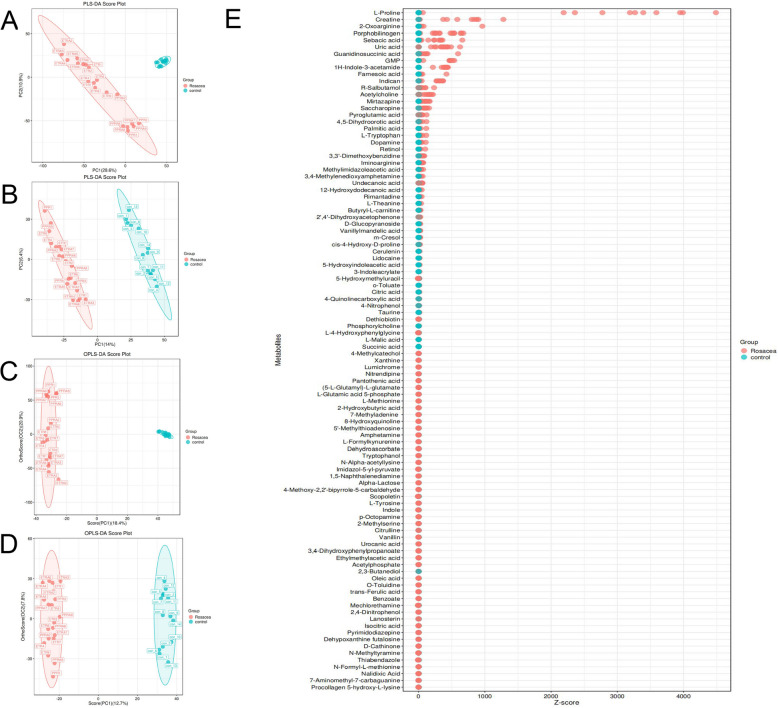
Multivariate Analysis and Metabolite Importance in Group Differentiation. **A** PLS-DA score plot in the positive ion mode; **B** PLS-DA score plot in the negative ionization mode; **C** OPLS-DA score plot in the positive ion mode; **D** OPLS-DA score plot in the negative ionization mode; E. Variable importance in projection (VIP) scores for the top 56 differentially abundant metabolites, ranked by their significance in group differentiation. PLS-DA, Partial Least Squares Discriminant Analysis; OPLS-DA, Orthogonal Partial Least Squares Discriminant Analysis; VIP, Variable Importance in Projection

Our results from both supervised, including PLS-DA (Fig. 1A, B) and OPLS-DA (Fig. 1C, D), and unsupervised analyses (i.e., PCA) (Figure S1A, B) revealed a proper separation of all patients with neurogenic rosacea from healthy controls in either positive or negative ionization mode, suggesting a significant alteration of the metabolic profile in the peripheral system of Chinese patients with neurogenic rosacea.

**Fig. 2:**
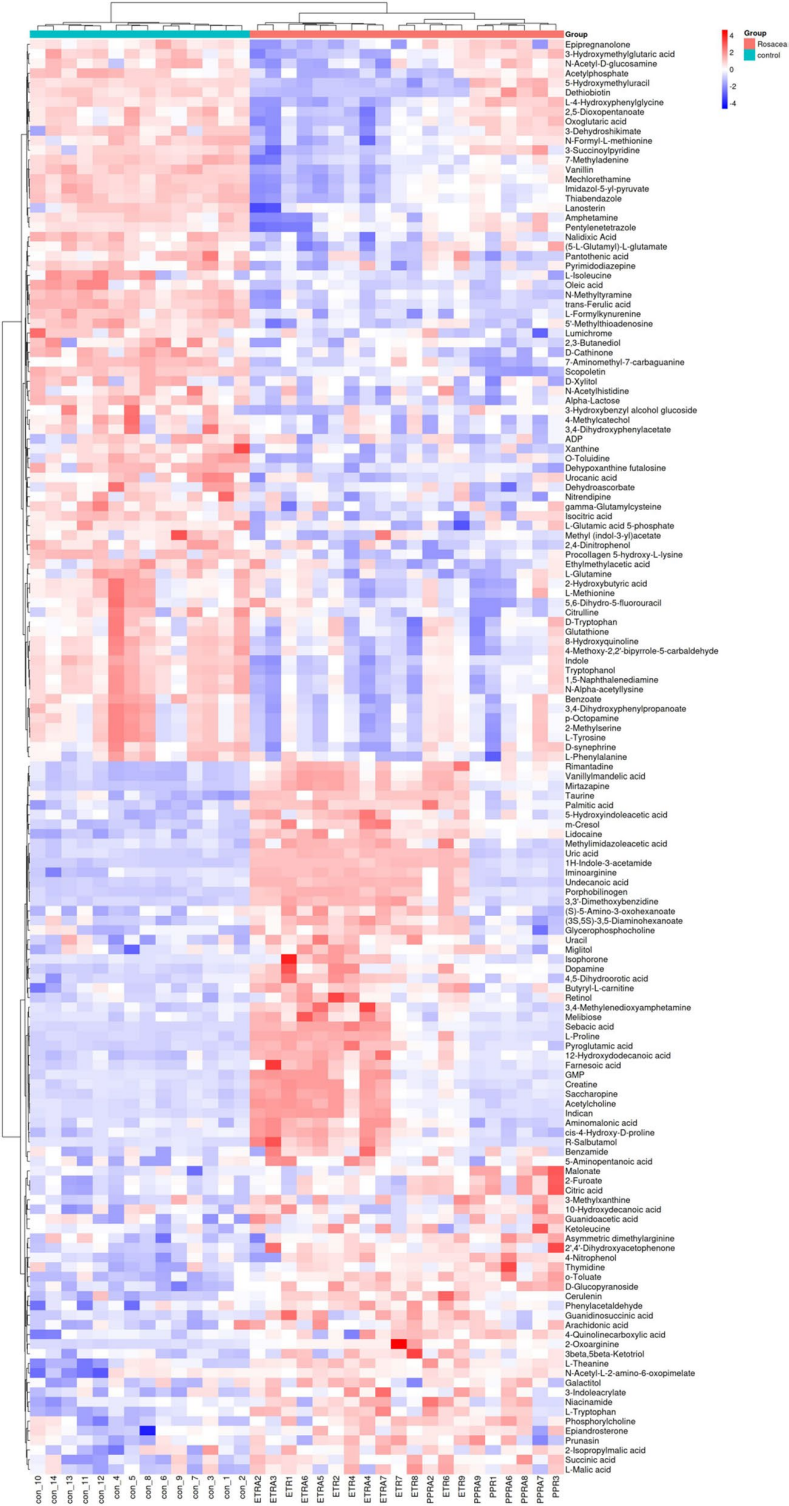
Distinct metabolic profiles of neurogenic rosacea patients and healthy controls. Heatmap depicting the relative abundance levels of all differentially abundant metabolites (DAMs) across the study groups, illustrating distinct metabolic profiles between neurogenic rosacea patients and healthy controls. DAMs, differentially abundant metabolites

**Fig. 3:**
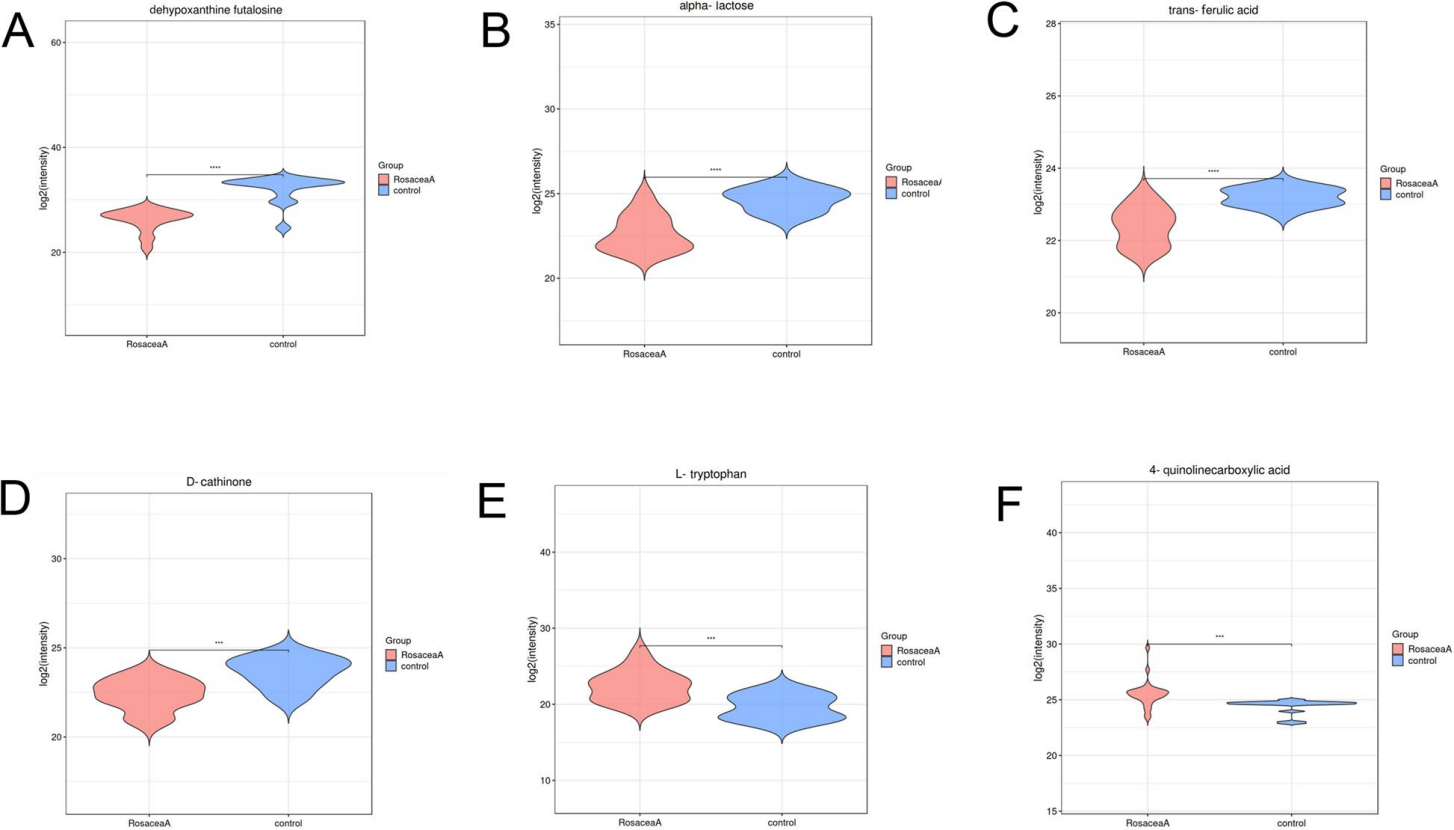
Representative metabolite differences between neurogenic rosacea patients and healthy controls. Boxplots showing the relative abundance of six representative metabolites with significant differences between neurogenic rosacea patients and healthy controls

Using LC–MS/MS analysis, we identified 149 differentially abundant metabolites (DAMs) in patients with neurogenic rosacea (Fig. 1E; Table S1 and S2). In addition, we found that the most populated category among DAMs was “Amino acids, peptides, and analogues” (22/149,14.8%), “Furoic acid and derivatives” (10/149, 6.7%), “Amines” (7/149, 4.7%), followed by “Carbohydrates and carbohydrate conjugates” (6/149, 4.0%), “Purines and purine derivatives” (4/149, 2.7%), and “Pyrimidines and pyrimidine derivatives” (3/149, 2.0%) (Figure S2). Further analysis revealed a significant increase and decrease in the relative levels of 74 and 75 differential metabolites, respectively, in patients with neurogenic rosacea as compared with healthy subjects (Fig. 2 and Table S1). Specifically, the top six metabolites ranked by VIP scores were o-toluate (VIP = 2.20, padj = 4.51e-6), D-glucopyranoside (VIP = 2.17, padj = 4.99e-6), Alpha-lactose (VIP = 2.16, padj = 9.90e-6), trans-ferulic acid (VIP = 2.12, padj = 1.82e-11), lidocaine (VIP = 2.01, padj = 6.65e-9), and L-formylkynurenine (VIP = 1.99, padj = 1.12e-8) (Fig. 3A-F).

### Microbial diversity and taxonomic differences

**Fig. 4:**
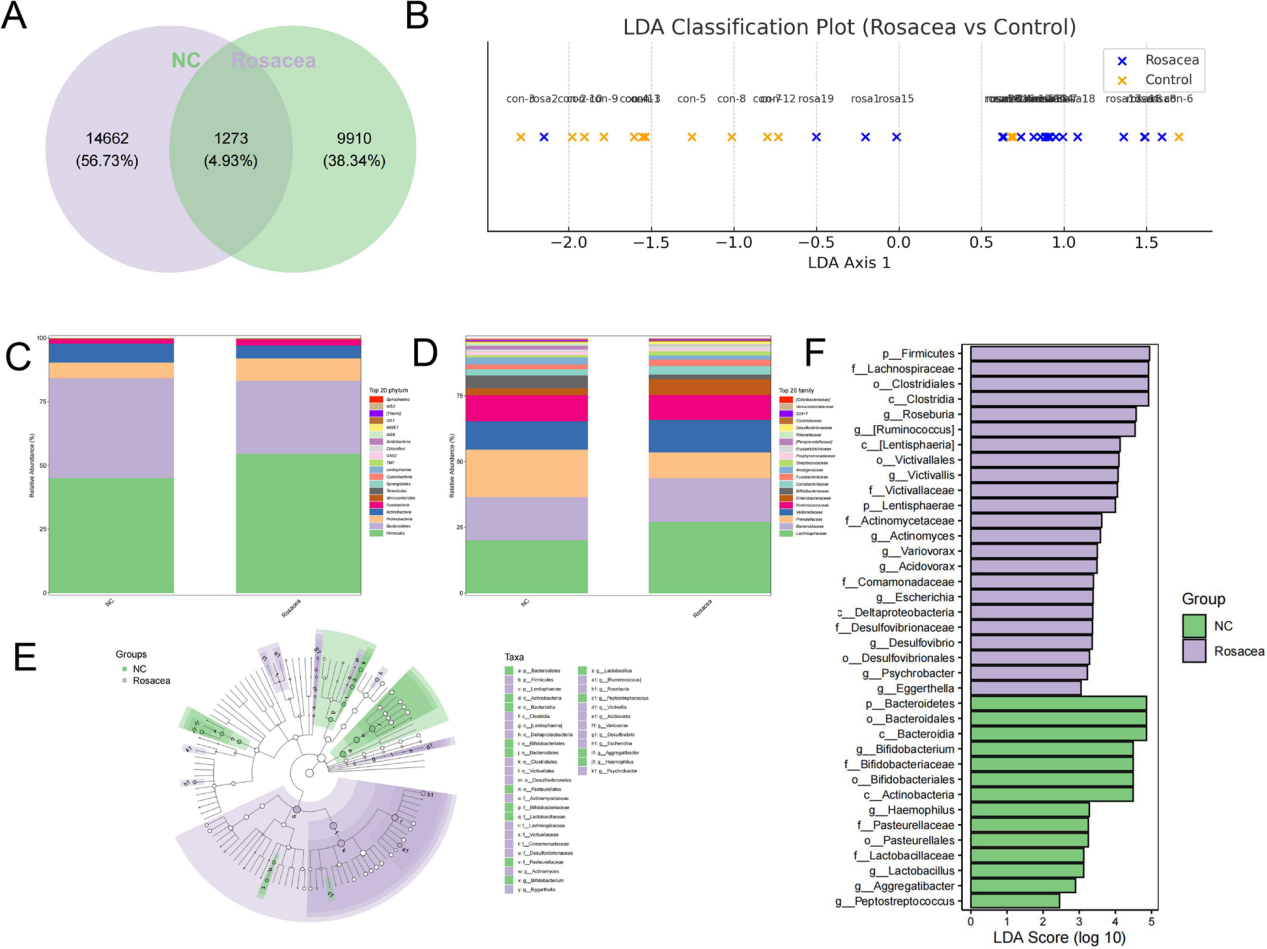
Comparative Gut Microbiota Analysis Between Neurogenic Rosacea Patients and Healthy Controls. Comparison of gut microbiota between neurogenic rosacea patients and healthy controls. **A** Venn diagram illustrating the average number of operational taxonomic units (OTUs) and overlapping OTUs in different groups. **B** Linear Discriminant Analysis (LDA) of microbial composition. Samples from the Rosacea group (blue) and the Control group (orange) are projected along the primary discriminant axis. Each dot represents an individual sample, labeled accordingly. **C** Microorganism community structures of the microbiota at the phylum level for each group. **D** Community bar plot analysis of microbiota composition at the family level in different groups. **E**, **F** Linear Discriminant Analysis Effect Size (LEfSe) analysis identifying differentially abundant taxa in the microbiota of the studied groups. OTUs, operational taxonomic units; PCoA, Principal Coordinate Analysis; NMDS, Non-metric Multidimensional Scaling; LEfSe, Linear Discriminant Analysis Effect Size

Analysis of taxonomic composition based on operational taxonomic units (OTUs) revealed 1273 microbial species that were commonly present in the fecal samples of both the neurogenic rosacea group and the healthy control group. (Fig. 4A). Linear Discriminant Analysis (LDA) revealed significant differences in microbial composition between the neurogenic rosacea group and healthy controls. The LDA projection showed a clear trend of separation along the primary discriminant axis, indicating structured distinctions in gut microbial profiles that may underly the pathophysiology of neurogenic rosacea (Fig. 4B).

Compared to the healthy control group, the neurogenic rosacea group exhibited a significantly higher abundance of *Firmicutes* and *Proteobacteria*, along with a notable reduction in *Bacteroidetes* and *Actinobacteria* at the phylum level (Fig. 4C). At the family level, the neurogenic rosacea group showed increased levels of *Lachnospiraceae*, *Veillonellaceae*, and *Clostridiaceae*, while the abundances of *Prevotellaceae* and *Bifidobacteriaceae* were significantly decreased (Fig. 4D).

Linear discriminant analysis (LDA) effect size (LEfSe) revealed distinct differences in microbial composition between the two groups. Specifically, the rosacea group exhibited an enrichment of several bacterial taxa, particularly from the *Firmicutes* and *Lentisphaerae* phyla. At the class level, the rosacea group was predominantly enriched with *Clostridia*, *Lentisphaeria*, and *Deltaproteobacteria*. The key bacterial orders identified in this group were *Clostridiales*, *Victivales*, and *Desulfovibrionales*. Further classification at the family level revealed a significant enrichment of *Lachnospiraceae*, *Victivallaceae*, *Actinomycetaceae*, *Comamonadaceae*, and *Desulfovibrionaceae*. At the genus level, bacteria such as *Roseburia*, *Ruminococcus*, *Victivallis*, *Actinomyces*, *Variovorax*, *Acidovorax*, *Escherichia*, *Desulfovibrio*, *Psychrobacter*, and *Eggerthella* were notably more abundant in the rosacea group. In contrast, the gut microbiota profile of the healthy control group was associated with a balanced and beneficial microbial community. This group was enriched with bacterial taxa belonging to the phyla *Bacteroidetes* and *Actinobacteria*. At the class level, the dominant taxa were *Bacteroidia* and *Actinobacteria*, with *Bacteroidales*, *Bifidobacteriales*, and *Pasteurellales* as key orders. At the family level, *Bifidobacteriaceae*, *Pasteurellaceae*, and *Lactobacillaceae* were particularly enriched. Genera, such as *Bifidobacterium*, *Haemophilus*, *Lactobacillus*, *Aggregatibacter*, and *Peptostreptococcus*, were significantly more abundant in the healthy group (Fig. 4E, F).

### Metabolite and intestinal microbiota correlation analysis

We subsequently investigated the potential correlations between the changes in metabolites and the spectra of the intestinal microbiome. Spearman’s correlation coefficient revealed significant relationships between the differences in intestinal flora and serum metabolites between the two groups (Table S2).

In terms of strong correlations, 2-oxoarginine exhibited a remarkably strong positive correlation with *Aggregatibacter* (r = 0.9099, padj = 6.31e-11). Additionally, 3,4-dihydroxyphenylacetate demonstrated a significant positive correlation with both *Bifidobacterium* (r = 0.7307, padj = 9.25e-07) and *Lactobacillus* (r = 0.6898, padj = 6.39e-06). Furthermore, rimantadine and 3-methylxanthine displayed strong positive correlations with *Psychrobacter*, with correlation coefficients of r = 0.7043 (padj = 7.82e-04) and r = 0.6849 (padj = 1.10e-03), respectively.

In terms of moderate correlations, several metabolites were positively correlated with *Ruminococcus*, including porphyrobilinogen (r = 0.6703, padj = 1.69e-03), vanillylmandelic acid (r = 0.6513, padj = 3.02e-03), and miglitol (r = 0.5832, padj = 2.45e-02), all of which were statistically significant. Regarding *Bifidobacterium*, positive correlations were observed with 8-hydroxyquinoline (r = 0.5810, padj = 2.45e-02), alpha-lactose (r = 0.5666, padj = 2.60e-02), and N-alpha-acetyllysine (r = 0.5589, padj = 2.95e-02). Additionally, alpha-lactose was also positively correlated with *Lactobacillus* (r = 0.5661, padj = 2.60e-02).

## Discussion

The current study provides compelling evidence of metabolic and microbial dysbiosis associated with neurogenic rosacea, revealing crucial insights into its pathophysiological mechanisms. Our findings demonstrated significant alterations in serum metabolomic profiles, with 149 differentially abundant metabolites (DAMs) identified via LC–MS/MS analysis. Notably, categories such as amino acids, peptides, and analogues, furoic acid and derivatives, and amines were predominantly enriched.

These findings suggest a profound disruption in metabolic pathways that may reflect the systemic neurogenic and inflammatory responses characteristic of rosacea [4, 5, 30].

Among the top-ranked metabolites, o-toluate, D-glucopyranoside, alpha-lactose, trans-ferulic acid, lidocaine, and L-formylkynurenine have emerged as potential biomarkers warranting further investigation. Alpha-lactose, a naturally occurring sugar in milk, has been studied for its immunomodulatory effects, influencing antigen-presenting cells (APCs) and T cell function, reducing oxidative stress in macrophages, and modulating costimulatory molecule expression. Additionally, it regulates T cell activation through an IL-2-dependent mechanism, exerting broader effects on immune responses [31]. D-glucopyranosides, such as those isolated from almond skins, exhibit strong antioxidant properties. Compounds like 3'-O-methylquercetin 3-O-β-D-glucopyranoside demonstrate significant free radical scavenging activity, which supports skin health by mitigating oxidative stress [32]. Ferulic acid functions as a potent antioxidant by neutralizing free radicals and inhibiting radical-generating enzymes, thereby protecting key skin structures, including keratinocytes and collagen, from oxidative damage [33, 34]. Lidocaine, widely used as a local anesthetic, has also demonstrated anti-inflammatory properties, suggesting its potential role in managing inflammatory conditions [35, 36]. L-formylkynurenine, a key intermediate in the kynurenine pathway, plays a crucial role in immune regulation and inflammation, contributing to both pro-inflammatory and tolerogenic processes by modulating cytokine production and immune cell function [37, 38]. Collectively, these bioactive compounds may contribute to the pathophysiology of rosacea through their effects on immune modulation, oxidative stress, and inflammatory signaling, underscoring their potential as mechanistic links or therapeutic targets.

Our analysis of the gut microbiota revealed profound differences in microbial composition and diversity between patients with neurogenic rosacea and healthy controls. The significant increase in *Firmicutes* and *Proteobacteria,* along with a decrease in *Bacteroidetes* and *Actinobacteria*, indicates a dysbiotic state that has been linked to various inflammatory diseases [39–41]. Enriched families, particularly *Lachnospiraceae* and *Clostridiaceae,* have been associated with inflammatory processes and metabolic syndromes [42–45], further implicating the gut-skin axis in the pathogenesis of neurogenic rosacea.

Correlation analysis revealed intriguing interdependencies between serum metabolites and the gut microbiota composition. Notably, the strong positive correlation between 2-oxoarginine and *Aggregatibacter* suggests a metabolic pathway that links gut-derived metabolites to systemic inflammatory responses. Previous studies indicate that 2-oxoarginine may serve as a potential pharmaceutical agent for the treatment of skin inflammatory diseases by modulating both pro-inflammatory and anti-inflammatory cytokines, regulating key signaling pathways, and targeting specific bacteria [46]. Our research further elucidated the association between 2-oxoarginine and *Aggregatibacter*, highlighting a possible mechanism that connects these metabolic and bacterial factors to inflammation. Additionally, previous studies have demonstrated that *Aggregatibacter* can induce the production of pro-inflammatory cytokines [47]. Taken together, these findings emphasize the crucial role of metabolic and bacterial interactions in regulating systemic inflammatory responses. Moreover, 3,4-dihydroxyphenylacetic acid (3,4-DHPA) possesses antioxidant properties and improved physicochemical characteristics, which can be beneficial for various biological processes [48]. In our study, 3,4-DHPA exhibited significant positive correlations with both *Bifidobacterium* and *Lactobacillus* in patients with neurogenic rosacea. This suggests that 3,4-DHPA may play a role in modulating the gut microbiota in these patients, possibly contributing to the metabolic and inflammatory dynamics of the disease through its antioxidant effects. *Ruminococcus* is known for its role in the metabolism of cellulose and complex carbohydrates, and is closely associated with gut health and immune function [49]. Porphobilinogen serves as a crucial intermediate in the heme biosynthesis pathway [50], while vanillylmandelic acid is the end product of catecholamine metabolism [51], and miglitol functions as an α-glucosidase inhibitor [52]. The positive correlations identified among porphobilinogen, vanillylmandelic acid, and miglitol suggest potential mechanisms by which gut microbiota may influence metabolic and neurogenic disorders. Furthermore, 8-hydroxyquinoline is a widely studied compound, known for its antioxidant and anti-inflammatory properties [53]. N-acetyl-L-lysine, an amino acid derivative, has been found to exhibit various biological activities, including modulation of immune responses and promotion of nerve growth factor synthesis [54]. In this study, we identified a positive correlation between 8-hydroxyquinoline, N-acetyl-L-lysine, and *Bifidobacterium*. This finding suggests that 8-hydroxyquinoline may potentially influence gut health through its antioxidant and anti-inflammatory effects, whereas N-acetyl-L-lysine may support immune function by promoting the growth of beneficial bacteria.

Additionally, to address limitations in unsupervised clustering methods, we explored t-distributed stochastic neighbor embedding (t-SNE), a nonlinear dimensionality reduction technique commonly used for microbiome data visualization [55]. Consistent with the NMDS and PCoA results, the t-SNE plot did not reveal clear group-level clustering (Figure S3). Given the subtle differences in microbial community structure, we further employed a supervised method—Linear Discriminant Analysis (LDA)—to enhance discriminative power. The LDA projection demonstrated a more pronounced separation between neurogenic rosacea patients and controls, suggesting that supervised modeling may be better suited for identifying group-specific microbial signatures in contexts where variance is moderate.

Despite the robustness of our findings, several limitations of this study must be acknowledged. The relatively small sample size, particularly within the neurogenic rosacea group, may have limited the generalizability of our results. Future studies should include a larger, more diverse population to validate these findings and explore potential confounding variables. Additionally, although our cross-sectional study design provides a snapshot of metabolic and microbial changes, longitudinal studies are necessary to establish causality and elucidate the temporal dynamics of these alterations.

Moreover, reliance on specific metabolite and microbial databases for identification and classification introduces an inherent bias. While we utilized well-established resources, variations in database completeness and accuracy may have influenced the reliability of our findings. Thus, future investigations may benefit from employing advanced techniques, such as untargeted metabolomics and metagenomic sequencing, to gain a more comprehensive understanding of the underlying biochemical and microbial landscapes associated with neurogenic rosacea.

In summary, our findings elucidate critical metabolic and microbial alterations in neurogenic rosacea and provide a comprehensive understanding of the underlying mechanisms. This integrative approach underscores the potential for developing targeted interventions that consider both metabolic and microbiota profiles, paving the way for novel therapeutic strategies aimed at restoring the microbial balance and modulating systemic inflammation in affected patients. Future longitudinal studies are warranted to establish causality and to further explore the therapeutic potential of microbiome modulation in managing neurogenic rosacea.

## Supplementary Information

Supplementary Material 1. Figure S1. Principal Component Analysis (PCA) of Metabolomic Profiles in Positive Ion Mode. A. PCA score plot for the positive ion model; B. PCA score plot in the positive ion mode. Figure S2. Classification of Differentially Abundant Metabolites (DAMs) by Chemical Categories. The distribution of differentially abundant metabolites (DAMs) across chemical categories in neurogenic rosacea patients and healthy controls. Figure S3. t-SNE visualization of gut microbiota composition in neurogenic rosacea and control groups. Each point represents a sample, with rosacea samples shown in blue and control samples in orange. t-distributed stochastic neighbor embedding (t-SNE) was applied to the standardized microbial abundance data to explore potential clustering patterns. No distinct group separation was observed, suggesting that group-level differences are subtle and not readily captured by this unsupervised nonlinear method.

Supplementary Material 2.Table S1. Intensity of metabolites significantly altered between patients with neurogenic rosacea and healthy controls (VIP ≥ 1.000, P value < 0.05). Table S2. Metabolite and Intestinal Microbiota correlation analysis

## Data Availability

The metabolomics data involved in this study have been submitted to the MetaboLights database under project number MTBLS11801 (https://www.ebi.ac.uk/metabolights/MTBLS11801). Additionally, the microbiome data associated with this study have been deposited in the NCBI Sequence Read Archive (SRA) under the accession number PRJNA1191396 (https://www.ncbi.nlm.nih.gov/bioproject/PRJNA1191396).

## Abbreviations

3,4-DHPA: 3,4-Dihydroxyphenylacetic Acid
APCs: Antigen-Presenting Cells
BMI: Body Mass Index
DAMs: Differentially Abundant Metabolites
EDTA: Ethylenediaminetetraacetic Acid
FC: Fold Change
HMDB: Human Metabolome Database
IL-2: Interleukin-2
KEGG: Kyoto Encyclopedia of Genes and Genomes
LDA: Linear Discriminant Analysis
LC–MS/MS: Liquid Chromatography-tandem Mass Spectrometry
LEfSe: Linear Discriminant Analysis Effect Size
LipidMaps: Lipid Metabolites and Pathways Strategy
MassBank: Mass Spectral Database
MTBLS: MetaboLights Study Identifier
NMDS: Non-metric Multidimensional Scaling
OPLS-DA: Orthogonal Partial Least Squares Discriminant Analysis
OTUs: Operational Taxonomic Units
padj: Adjusted p-value)
PCA: Principal Component Analysis
PCoA: Principal Coordinate Analysis
PCR: Polymerase Chain Reaction
PLS-DA: Partial Least Squares Discriminant Analysis
PRJNA: NCBI BioProject Identifier
QIIME: Quantitative Insights Into Microbial Ecology
QC: Quality Control
RSD: Relative Standard Deviation
VIP: Variable Importance in Projection
