# Supplementary figures and images for: Correlations between gut microbiota and serum metabolomics in patients with neurogenic rosacea
Source: BMC Microbiol. 2025 Jul 17;25:441. doi: 10.1186/s12866-025-04173-3 (PMC12272963; doi:10.1186/s12866-025-04173-3)

**Fig. S1**


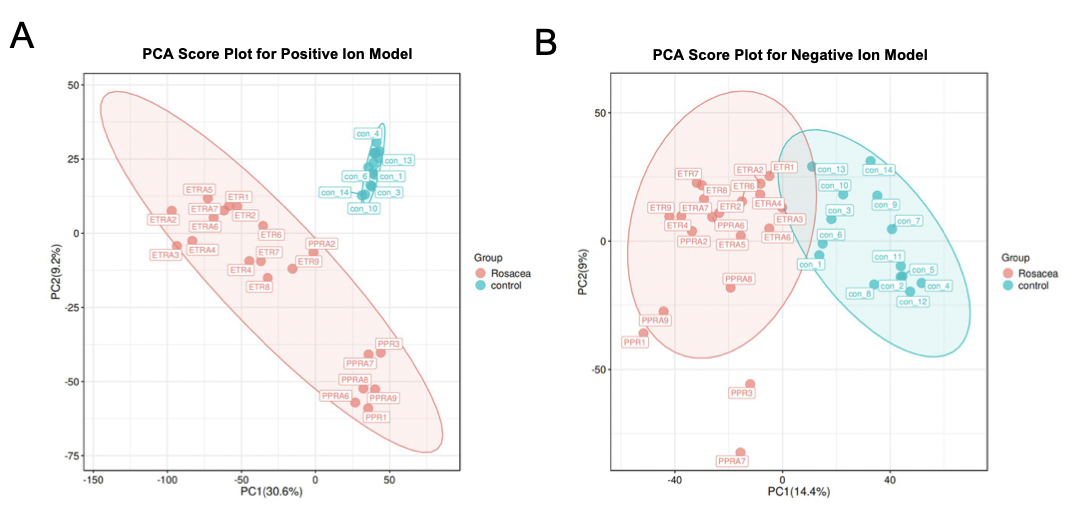


**Fig. S2**


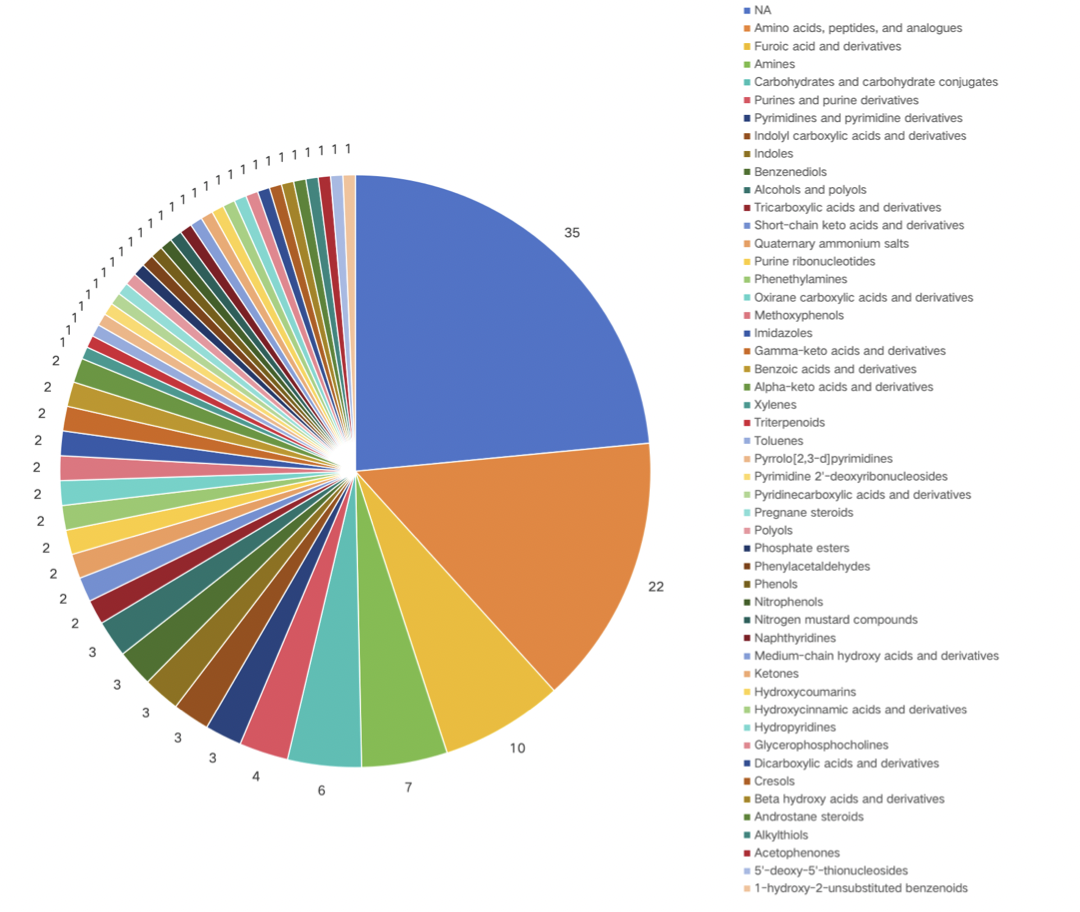


**Fig. S3**

**
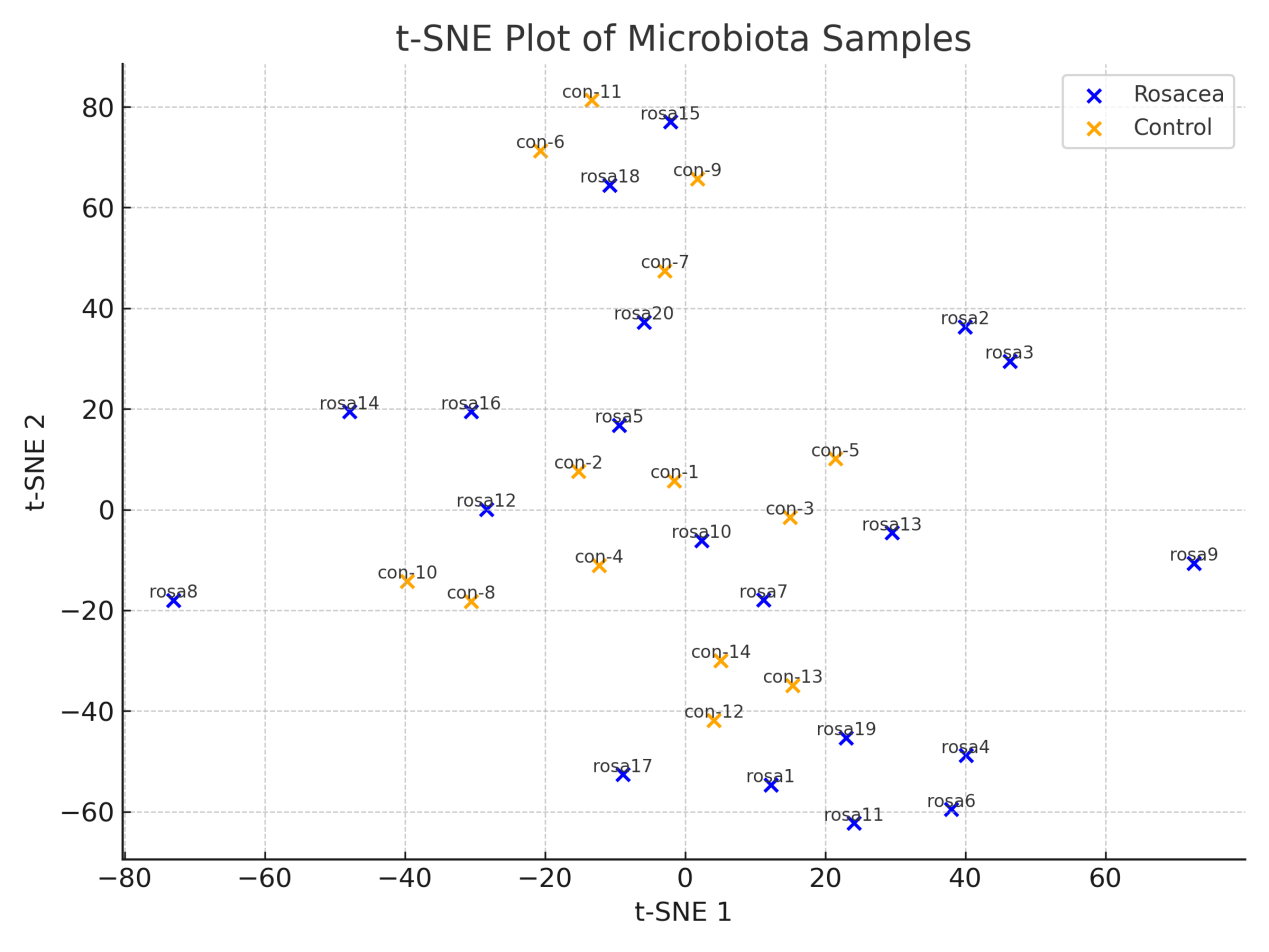
**

Supplement: Supplementary file 1 — Supplementary Material 1. Figure S1. Principal Component Analysis (PCA) of Metabolomic Profiles in Positive Ion Mode. A. PCA score plot for the positive ion model; B. PCA score plot in the positive ion mode. Figure S2. Classification of Differentially Abundant Metabolites (DAMs) by Chemical Categories. The distribution of differentially abundant metabolites (DAMs) across chemical categories in neurogenic rosacea patients and healthy controls. Figure S3. t-SNE visualization of gut microbiota composition in neurogenic rosacea and control groups. Each point represents a sample, with rosacea samples shown in blue and control samples in orange. t-distributed stochastic neighbor embedding (t-SNE) was applied to the standardized microbial abundance data to explore potential clustering patterns. No distinct group separation was observed, suggesting that group-level differences are subtle and not readily captured by this unsupervised nonlinear method. [file 12866_2025_4173_MOESM1_ESM.docx]
